# Immunophenotyping, activation, and expansion of gamma delta T cells: flow cytometry profiling and *ex vivo* manipulation strategies

**DOI:** 10.3389/fimmu.2026.1829178

**Published:** 2026-07-31

**Authors:** Urška Rupnik, Pia Bertoncelj, Katja Leben Zupet, Gašper Markelj, Tina Vesel Tajnšek, Neja Šamec, Tadej Avčin, Jelka Pohar, Anže Smole

**Affiliations:** 1 Immunology and Cellular Immunotherapy Group, Department of Genetic Toxicology and Cancer Biology, National Institute of Biology, Ljubljana, Slovenia; 2 Department of Allergology, Rheumatology and Clinical Immunology, University Children’s Hospital, University Medical Center Ljubljana, Ljubljana, Slovenia; 3 Clinical Institute of Special Laboratory Diagnostics, University Children’s Hospital, University Medical Center Ljubljana, Ljubljana, Slovenia; 4 Faculty of Medicine, University of Ljubljana, Ljubljana, Slovenia; 5 Biotechnical Faculty, University of Ljubljana, Ljubljana, Slovenia

**Keywords:** barrier immunity, cellular immunotherapy, flow cytometry, gamma delta (γδ) T cells, immune dysregulation, immunophenotyping, γδ T cell activation, γδ T cell expansion

## Abstract

Gamma delta (γδ) T cells exhibit unique antiviral, antibacterial, and antitumor properties. Advancing the fundamental understanding of γδ T cell immunobiology and unlocking their full therapeutic potential requires detailed immunophenotypic profiling alongside optimized strategies for *ex vivo* activation and expansion. In this review, we synthesize current knowledge of γδ T cell immunobiology, emphasizing (i) comprehensive phenotypic characterization using flow cytometry and (ii) emerging approaches for their therapeutic implementation, with a focus on activation and *ex vivo* expansion methods. By integrating insights from phenotypic analysis with a comparative evaluation of expansion techniques, we provide an overview of γδ T cell immunobiology and their translational potential in immunotherapy.

## 1 Introduction

The immune system comprises a highly diverse array of immune cells, each playing a specific role in mediating immune responses (1). At the intersection of adaptive and innate immunity are gamma delta (γδ) T cells, whose unique features are both biologically fascinating and therapeutically intriguing (2). Discovered in the mid-1980s, γδ T cells are T lymphocytes whose TCR consists of γδ rather than αβ chains (3–5). They exhibit unique properties, such as antiviral, antibacterial, and antitumor activity (6). γδ T cells combine functionalities of T cells, NK cells, and antigen-presenting cells, and show tissue tropism specific to certain subtypes (6). They are a heterogeneous population with complex TCR repertoires, recognizing various antigens, including proteins and lipids (7). Vγ9Vδ2 T cells, the predominant subset in peripheral blood (6, 8), recognize small, phosphorylated metabolites called phosphoantigens (pAgs) through their semi-invariant TCRs (6). pAgs are upregulated under stress conditions including malignant transformations and infections (9). Another two major subsets of γδ T cells characterized by adaptive-like immunobiology and more diverse TCR repertoires are Vδ1 and Vδ3 T cells. Due to their unique properties, including the capacity of some γδ T cell products to take up and cross present the processed tumor-derived antigens in experimental setting (10), γδ T cells have proven to be a highly promising modality for cancer immunotherapy in the context of γδ CAR (chimeric antigen receptor)-T cells (10–14). Given their semi-invariant TCR repertoire and MHC-independent antitumor activity, they hold potential for allogeneic “off-the-shelf” cellular products (12).

Beyond cancer and infection, γδ T cells contribute to immune homeostasis and tissue integrity and can participate in immune dysregulation when their effector programs become imbalanced. Across inflammatory and immune-mediated conditions, distinct γδ subsets may adopt pro-inflammatory (e.g., IL-17/IL-22) or immunoregulatory (e.g., IL-10/TGF-β) programs, with outcomes that are context dependent and shaped by local cues (15, 16). In disorders such as rheumatoid arthritis and multiple sclerosis, IL-17-producing γδ T cell subsets are frequently associated with chronic inflammation, synovial activation, and tissue injury (16, 17). These cells can enhance macrophage activation, fibroblast proliferation, and osteoclastogenesis, contributing to structural damage. At the same time, regulatory γδ T cells secreting IL-10 and TGF-β may counterbalance excessive autoimmune responses, reflecting the functional plasticity of this immune population (16). In inborn errors of immunity, quantitative and qualitative alterations of γδ T cells have been described. In this setting, γδ T cells may serve as an auxiliary defense mechanism when adaptive immunity is compromised, contributing to early antimicrobial responses through rapid effector functions, including IFN-γ and IL-17 production (18). Dysfunctional γδ T cell development or signaling may increase susceptibility to recurrent infections and impair immune surveillance. Studies on innate-like lymphocytes suggest that γδ T cell deficiency may correlate with more severe clinical phenotypes in selected inborn errors of immunity conditions (18). Barrier diseases represent another major functional domain of γδ T cells. In asthma, γδ T cells participate in airway immune regulation by modulating Th2 and Th17 responses. IL-17-producing subsets may promote neutrophilic inflammation and bronchial hyperresponsiveness, particularly in severe or steroid-resistant asthma phenotypes. Similarly, in inflammatory bowel disease, especially Crohn’s disease, intraepithelial γδ T cells contribute to mucosal barrier integrity, microbial homeostasis, and epithelial repair. Loss of functional intraepithelial γδ T cells may exacerbate chronic intestinal inflammation (19–21). The role of γδ T cells is well studied in atopic dermatitis where they participate in both acute and chronic inflammation by producing cytokines such as IL-17, IL-22, and IFN-γ, which regulate keratinocyte proliferation, antimicrobial defense, and immune activation. While these cytokines support barrier protection, excessive or prolonged activity may promote chronic inflammation. γδ T cells also contribute to epithelial repair and microbiota regulation. Imbalance between pro-inflammatory and reparative functions may influence atopic dermatitis severity and recurrence (22, 23). The diverse roles of γδ T cells across these disease settings highlight their value as biomarkers in immune-mediated diseases. In addition to providing biological context, these settings illustrate why the study of γδ T cells is relevant both for fundamental and translational research and for clinical application, including the analysis of patient samples to support diagnosis, disease monitoring, and treatment-related decision-making.

Beyond these disease-oriented applications, continued research in γδ T cell immunobiology is also essential for unlocking their therapeutic potential. In this context, an in-depth understanding of γδ T cell biology, together with detailed phenotypic and functional characterization and advanced *ex vivo* manipulation approaches including activation, expansion, and genetic engineering is critical for the development of γδ T cell-based cellular immunotherapy strategies and for generating defined, functional cell products.

In this mini-review, we synthesize existing knowledge across two interconnected and complementary aspects of γδ T cell research: (i) a framework for flow-cytometric immunophenotyping, including the selection and interpretation of relevant markers, sample-type considerations, gating logic, and technical controls; and (ii) a comparative synthesis of *ex vivo* activation and expansion strategies, with emphasis on how protocol choices influence subset selectivity, differentiation trajectories, and suitability for downstream genetic engineering. By bringing these perspectives together, we highlight practical links between flow-cytometric readouts, manipulation strategies, and the desired properties of the final γδ T cell product, thereby providing a clearer framework for both biological investigation and translational application.

## 2 Immunobiology and immunoprofiling of γδ T cells via flow cytometry

γδ T cells are a rare T cell subtype comprising 1-10% of peripheral blood CD3^+^ T cell population in healthy humans (15, 24). They are predominately (>70%) CD4^−^CD8^−^ (double negative), some (30%) are CD8^+^CD4^−^ and very few (<1%) are CD4^+^CD8^−^ (25). These diverse populations differ in tissue homing as well as function (26). Human γδ T cells are a heterogenous population of subsets that express a combination of one of the 7 different Vγ TCR chains paired with one of the 4 Vδ TCR chains (27). Vδ2 is almost exclusively paired with Vγ9 and the resulting Vγ9Vδ2 T subtype represents the predominant subset (60-95%) in peripheral blood and lymphoid organs. This subtype participates in defense against malignant cells and has the ability to function as an antigen presenting cell (APC) (6, 8). Vδ1 cells are enriched in barrier tissues such as the gut mucosa, skin, and lungs, where they contribute to local immune defense by promoting recruitment of Th17 cells and neutrophils and by supporting epithelial antimicrobial responses (17). Vδ3 subtype is mainly found in the liver and intestines (28). The entire repertoire of γδ T cells is still unknown (17). Compared to conventional αβ T cells, that require recognition via TCR, engagement of costimulatory molecules and cytokine signaling for successful activation, the activation of γδ T cells is less understood. γδ T cells also express TCR that is required for antigen recognition, but they do not engage MHC-antigen complexes (29).

### 2.1 Characteristics and functions of γδ T cells

Bridging innate and adaptive immune systems, γδ T cells are endowed with antitumor mechanisms characteristic of αβ T cells, mediated by TCR and co-stimulatory signals, and NK cells, mediated by activating NK cell receptors (2). This diverse array of functions is the reason γδ T cells play fascinating and context−dependent roles in the immune response (17). Depending on the immunological context, γδ T cells can promote inflammation and recruitment of immune cells to site of infection or help in tissue repair and regulation of immune response. Most γδ T cells have antitumor activity, but some subsets also have pro-tumor properties in specific context (6).γδ T cells interact with other innate and adaptive immune cells, recruiting them to sites of infection and modulating their function through cytokine and chemokine secretion as well as antigen presentation. In this way, distinct γδ T-cell subsets can either promote inflammatory myeloid responses or support regulatory, tissue-repair-associated programs, depending on the local microenvironment (16). All these functionalities make characterizing the intrinsic features that distinguish specific subsets challenging (28).

The TCR of γδ T cells is composed of γ and δ chains, arranged through somatic recombination within the thymus similar to αβ T cells (28) and encoded by variable (V), diversity (D; only within delta chains) and joining (J) fragments (30). Although γδ T cells have the potential for extensive diversity, many mature subsets show biased Vγ/Vδ usage, leading to subset−specific repertoire patterns (16, 31). Although their genetic TCR profile is similar in principle to that of αβ TCRs, the recognition of activating ligands by γδ T cells is diverse and different from αβ TCRs. Particularly, γδ TCRs do not require MHC-mediated antigen presentation (32).

γδ T cells express some molecules related to NK cells like NK cell receptor NKG2D and other molecules that participate in enhanced tumor recognition such as FcγRIIIa (CD16a), DNAM-1, NKp46, NKp44 and NKp30 (6, 27). While γδ T cells do not recognize MHC class I-antigen complexes, they recognize MHC class I related molecules, MICA and MICB, stimulatory ligands of NKG2D (29). While some molecules like γδ TCR and NKG2D are characteristically expressed by all γδ T cells, some are subtype specific. Depending on inflammatory signals present in the tissue microenvironment γδ T cells can display a Th1, Th2, Th17 or Treg-like phenotype (17). Certain subsets have the ability to secrete interferon γ (IFN-γ), tumor necrosis factor α (TNF-α), proinflammatory chemokines CCL5 and CXCL10, and interleukins including IL-2 IL-4, IL-5, IL-13, IL-15 and IL-17. Interestingly, IL-17 production by γδ T cells has a distinct advantage over IL-17 production by αβ T cells as its activation in γδ T cells does not require antigen presentation by MHC I and results in more potent IL-17 concentration earlier in the immune response. When activated, γδ T cells exhibit strong cytotoxic activity through the release of granzyme B and perforin, by membrane bound TRAIL and Fas ligands and production of IFN-γ and TNF-α (33). γδ T cells located in the skin (mostly Vδ1) constitutively express the chemokine receptor CCR6 and the transcription factor RORγδ (17, 28). Based on their phenotype, γδ T cells are divided into four main differentiation subsets (naïve, central-memory, effector-memory or terminally differentiated) based on the expression of CD45RO or CD45RA in a combination with CD27, CCR7 or CD62L similarly to αβ T cells (34).

### 2.2 Characterization of γδ T cells via flow cytometry

**Figure 1:**
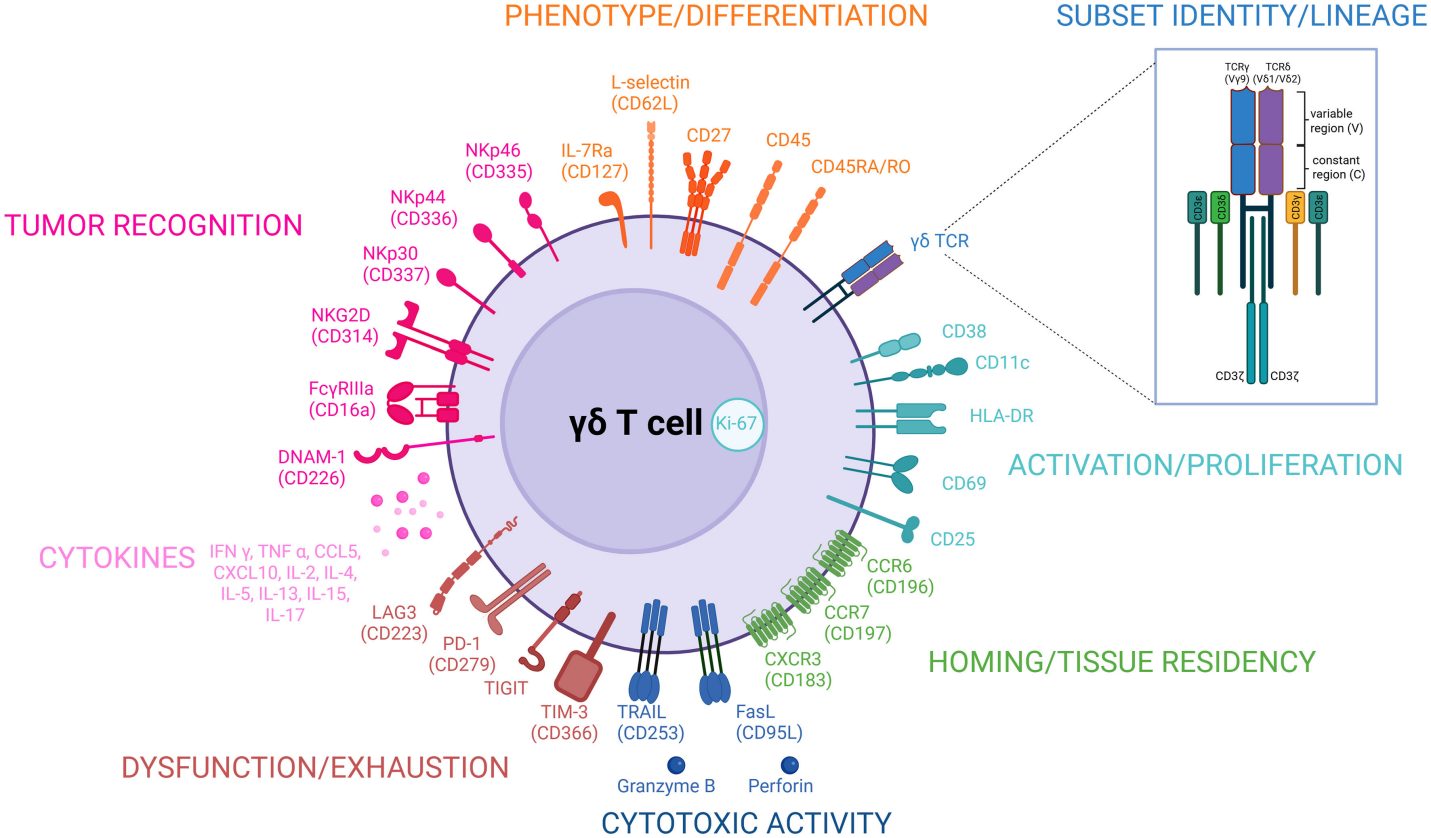
Schematic representation of a γδ T cell displaying key markers and cytokine secretion. The depicted markers are grouped into the analytical categories that capture certain elements of γδ T cell immunobiology, highlighting major surface markers and selected intracellular marker reflecting phenotypic/differentiation status, subset identity/lineage, activation/proliferation, homing/tissue residency, cytotoxic activity, dysfunction/exhaustion and tumor recognition. Upon stimulation, γδ T cells can release a range of cytokines representing common effector programs and mediating broader immune cross-talk. For clarity, markers are assigned to color-coded categories, although several markers have context-dependent functions and may belong to more than one analytical module. Created with BioRender. https://BioRender.com/xczkkhh.

In research and diagnostic settings, comprehensive immunophenotypic characterization encompassing subset composition, activation and differentiation states, functional and dysfunctional signatures, migratory and tissue−resident programs, and effector capacities is essential for resolving the complexity of immune responses (35). This requirement is particularly pronounced for γδ T cells, whose unconventional antigen−recognition modalities, tissue−restricted subset distributions, and context−dependent effector programs necessitate high−resolution profiling. **Figure 1** provides a schematic representation that captures certain elements of γδ T cell immunobiology, highlighting major surface markers and selected intracellular marker reflecting phenotypic/differentiation status, subset identity/lineage, activation/proliferation, homing/tissue residency, cytotoxic activity, dysfunction/exhaustion and tumor recognition as well as secreted cytokines. These markers delineate lineage identity and differentiation trajectories, govern activation thresholds and cytotoxic mechanisms, and contribute to recognition of malignant or stressed cells (6, 7, 28). In parallel, the cytokines released upon stimulation illustrate the integration of γδ T cells into broader immune−signaling networks and their roles in shaping effector responses. Collectively, such marker−level insights enable precise delineation of γδ T cell behavior across physiological and pathological contexts.

This information can provide insight into the characteristics and course of the disease and contribute to the development of better diagnostic methods and treatment strategies. Flow cytometry (FC) is an indispensable technology that allows us to characterize and evaluate individual cells or particles in a solution/suspension (36). The advancement of spectral FC allows for the detection of numerous factors in a sample, as well as deep immune profiling and characterization of various immune cells in mixed populations (37–39).

Depending on the type of FC available and the characteristics of the starting material, there is a plethora of possible Optimized Multicolor Immunophenotyping Panels (OMIPs) that can help select the necessary markers for the desired characterization of γδ T cells. Cossarizza et al. (2021) provide a detailed and effective starting point for the analysis of γδ T cells, starting with the preparation of peripheral blood sample, followed by FC using the BD LSRFortessa™ X-20 Cell Analyzer, with 12-color surface and intracellular marker staining. Their gating strategy includes the gating of lymphocytes, singlets, CD3+ cells, and TCRγδ+ T cells. This approach further enables the differentiation of γδ T cell subsets based on the expression of distinct γ and δ chains, enabling a more detailed phenotypic analysis (35). A comprehensive review of original articles and OMIPs reveals several notable advances in multicolor flow cytometry panels. Hammerich et al. (40) utilized a 31-color panel on a 3-laser Cytek^®^ Aurora cytometer to analyze human peripheral blood mononuclear cells (PBMCs). Although their panel was not specifically designed for γδ T cells, it is well suited for analyzing various T cell subtypes in human blood samples. Similarly, Liechti et al. (41) provided a comprehensive 30-parameter panel for immunophenotyping on a 5-laser FACSymphony A5, which enables comprehensive characterization of conventional and unconventional CD3+ T cell populations, including iNKT, MAIT and γδ T cells among others. Hertoghs et al. (42) presented a 27-color panel using a 5-laser FACSymphony A5 to detect multiple lymphoid cell subsets, including MAIT and γδ T cells. Park et al. (43) introduced a 40-color flow cytometry panel for immunophenotyping of all major cell subsets in human peripheral blood samples, utilizing a 5-laser Aurora Full Spectrum Cytometer. Finally, Anderson et al. (44) developed a 27-color flow cytometry panel on the same cytometer platform focusing on the phenotypic and functional characterization of both conventional and unconventional T cell populations. Focusing on T cell populations, Swanson et al. (45) described a 28-color FC panel that includes αβ and γδ T cell subsets, regulatory T cells (Tregs), T follicular helper cells (Tfh), and recent thymic emigrants (RTEs), with additional markers to measure cell surface activation molecules using a 5-laser FACSymphony A5. Similarly, Wang et al. (46) presented a 31-parameter panel for profiling T cell subsets based on surface markers, including iNKT, γδ T, MAIT, and Th cell subsets, as well as Tfh and Th cells with specific homing capabilities, also using a 5-laser FACSymphony A5. Finally, Barros-Martins et al. (47) provided a comprehensive 28-color flow cytometry analysis of the development and dynamics of γδ T cells in a clinical cohort. This full-spectrum panel, using a 5-laser Cytek^®^ Aurora system also included surface receptors to distinguish different stages of cell maturation, activating receptors, chemokine receptors, and NK-associated markers. Various OMIPs employ commonly used antibodies to define γδ T cell lineages by flow cytometry. These typically include pan γδ TCR antibodies and, more specifically, reagents for identifying the major γδ T cell subsets Vδ1 and Vδ2, whereas Vδ3 specific antibodies are rarely incorporated into flow cytometry panels. Among γ chain specific reagents, TCR Vγ9 is the most frequently used, while TCR Vα7.2 is routinely included when panels also profile the αβ T cell compartment. In the absence of specific antibodies characterization of γδ T-cell subtypes expressing alternative γ and δ chains has largely relied on single-cell transcriptomic analysis using RNA sequencing (48–51).

Effective and accurate identification of γδ T cells within complex samples, along with robust functional and phenotypic characterization, is essential for translating their unique immunobiology into therapeutic applications. This is particularly relevant in the context of cellular immunotherapy, where recent advances in the isolation, culturing, activation, and engineering of γδ T cells continue to expand their clinical potential (12).

#### 2.2.1 Practical workflow for γδ T cell flow cytometry

A γδ-focused analysis benefits from a structured workflow that separates (i) lineage identification, (ii) subset resolution, and (iii) functional-state readouts. Independent of instrument class, a typical pipeline includes sample selection and preprocessing, doublet exclusion, viability, hierarchical gating to define γδ T cells, and marker-based subsetting and functional interpretation.

Sample type also needs to be considered. Whole blood, especially in clinical protocols, enables rapid assessment with minimal manipulation and prevents loss of target population (e.g. blasts, granulocytes, some activated lymphocytes which can be lost upon gradient centrifugation) but requires careful red blood cell lysis and is less suited for extensive intracellular readouts. Although not impossible, incubation times in fixation and permeabilization buffers must be followed strictly (39, 40). Wang et al. observed differences in the distribution of innate-like T-cell subsets between whole-blood and PBMC staining, reporting a higher frequency of γδ T cells in PBMC-derived samples (46). Preprocessing whole blood using gradient centrifugation is commonly used to enrich for PBMCs, which enable standardized stimulation assays and intracellular staining but may underrepresent tissue-resident programs and are sensitive to cryopreservation effects (39). While cryopreserved PBMC samples are widely used in flow cytometry studies (41, 42, 44, 45, 47, 51) and offer the logistical advantage of enabling batched analysis of samples collected from multiple participants, cryopreservation can affect the expression of certain surface markers, including CD62L and various chemokine receptors (39, 46). In addition, cryopreservation has been reported to slightly reduce the resolution of TCRγδ detection, potentially impacting the accurate identification of γδ T-cell populations (46). Tissue-derived single-cell suspensions capture local γδ biology but introduce additional variables such as enzymatic digestion artifacts, increased autofluorescence, and lower cell yields, necessitating stringent viability control and panel optimization (52).

Regarding core gating logic, we recommend sequential gating on lymphocytes (FSC/SSC), singlets (FSC-A vs FSC-H and SSC-A vs SSC-H), live cells (fixable viability dye), CD45+ leukocytes, CD3+ T cells, and then TCRγδ+ events (40, 43, 45, 46). Subset resolution can then be performed using Vδ1 and Vδ2 (and Vγ9 where relevant) (41, 42, 47). During panel optimization, Hertoghs et al. observed a population of Vδ2^+^ cells that lacked staining for the pan-γδ TCR marker, suggesting potential epitope competition between the Vδ2 and TCRγδ antibodies. Matrix titration experiments indicated that a lower concentration of the Vδ2 antibody combined with a higher concentration of the TCRγδ antibody yielded the highest proportion of Vδ2^+^TCRγδ^+^ cells (42). Park et al. observed that sequential staining of TCRγδ, in which the anti-TCRγδ antibody (clone B1.1) was added separately rather than as part of the full staining cocktail, improved staining quality and resolution, thereby enabling more reliable identification of TCRγδ+ T cells (43).

Controls and QC are a foundation for robust and reliable FC analysis. High-dimensional panels require appropriate compensation or spectral unmixing controls, fluorescence-minus-one controls for low-density markers, and batch-aware normalization when comparing donors or longitudinal samples. Reporting should specify sample type (fresh vs thawed), stimulation conditions, and the minimal gating and control set used to support each conclusion. The design of a comprehensive multiparametric flow cytometry panel for γδ T cell analysis should be guided by the underlying biological question and accompanied by rigorous assay optimization. This process should include appropriate controls as well as evaluation of fluorochrome combinations (41, 42, 44, 47, 53), antibody clones and reagent suppliers (43–47), and antibody concentrations (41, 43–46) to ensure optimal signal resolution and accurate population identification. While surface staining enables rapid sample processing and preserves cell viability for downstream applications (39), the inclusion of intracellular staining substantially expands the scope of phenotypic and functional characterization. Intracellular markers can be used to assess cytokine production (45, 54, 55), costimulatory molecules (e.g. CD40L (45)), transcription factors (54, 55), proliferation markers such as Ki-67 (42), adaptor proteins (e.g., FcϵRγ (42)), and cytotoxic effector molecules including granzymes and perforin (44). Collectively, these approaches enable a more comprehensive characterization of γδ T cell differentiation, activation, and effector function, while also helping to define their role within the broader immune landscape.

The number of fluorophores included in a flow cytometry panel should be determined by the capabilities of the available platform, whether conventional or spectral. Spectral flow cytometers and other high-parameter analyzers support larger fluorophore panels, enabling simultaneous characterization of multiple cell types (41–43, 45, 46) or detailed analysis of multiple markers within a single population of interest. For γδ T cells, this allows high-resolution assessment of phenotypic, functional, and differentiation heterogeneity (47, 51, 53). Additionally, high-parameter approaches reduce sample requirements, an important advantage when working with patient material, while also lowering experimental complexity and enabling detailed immune profiling.

Comprehensive full-spectrum flow cytometry panels for analyzing human γδ T-cell subsets have been described by Barros-Martins et al. (47) and Tan et al. (51) as validated tools for detailed phenotypic and functional characterization of these cells, advancing understanding of their roles in immunity and disease. A key strength of these panels is their ability to capture the phenotypic and functional diversity of human γδ T-cell subsets identified by single-cell RNA sequencing and TCR sequencing (51). They are also well suited for longitudinal and exploratory studies of human γδ T cells in blood and cord blood from clinical cohorts (47). This panel combines TCR-specific antibodies with surface markers for lineage, activation, differentiation, memory, and trafficking to enable detailed characterization of γδ T-cell subsets, including Vγ9^+^Vδ2^+^, Vδ1^+^, and Vδ3^+^ populations, while also identifies MAIT cells through TCR Vα7.2. It supports analysis of maturation states ranging from naïve and central memory to effector memory and terminally differentiated cells, using differentiation markers such as CD45RA, CD27, CD28, CD127, CD57, and CD16; activation and effector markers including CD69, CD161, CD56, NKG2A, NKG2D, PD-1, CD38, and CD25; and chemokine receptors such as CXCR6, CCR5, CCR6, and CX3CR1. These features facilitate high-dimensional phenotypic mapping using dimensionality reduction tools such as UMAP and t-SNE (47).

#### 2.2.2 Marker framework for γδ T cell immunophenotyping

For interpretability and cross-study comparability, markers can be grouped by analytical purpose into subset identity/lineage, phenotype/differentiation, activation and proliferation, dysfunction/exhaustion, homing and tissue residency, and cytotoxicity/tumor-recognition modules (**Table 1**; a surface-marker subset is illustrated in **Figure 1**). This organization supports panel design aligned with the biological question and helps translate phenotypes into expectations for *ex vivo* expansion and final product properties.

**Table 1:** Marker framework for γδ T cell immunophenotyping.

Analytical category	Representative markers	Typical interpretation/use
Subset identity/lineage	CD3, TCRγδ, Vδ1, Vδ2, (Vγ9)	Define γδ T cells and resolve major circulating subsets; interpret expected responsiveness to phosphoantigen/bisphosphonate stimulation.
Phenotype/Differentiation	CD45RA/RO, CD27, CCR7, CD62L, CD127, CD28	Estimate naïve/central-memory/effector-memory/terminal differentiation; informs persistence potential and culture-induced differentiation drift.
Activation/proliferation	CD69, CD25, HLA-DR, CD11c, CD38, Ki-67	Quantify activation state and recent proliferation; supports monitoring of stimulation intensity during ex vivo manipulations.
Dysfunction/exhaustion	PD-1 (CD279), TIM-3 (CD366), LAG-3 (CD223), TIGIT, CD39 (panel dependent)	Detect chronic stimulation signatures; supports assessment of product quality and need for protocol adjustment. Need to be careful with interpretation and distinction between exhaustion/dysfunction and activation
Homing/tissue residency	CCR6, CXCR3, CCR5, CXCR6, α4β7, CLA, CD103, CD49a, CD69	Infer trafficking programs and tissue residency; relevant when comparing PBMC vs tissue-derived γδ T cells and for therapeutic targeting.
Cytotoxicity/tumor recognition	Granzyme B, Perforin, CD107a, NKG2D, DNAM-1, NKp30/44/46, CD16	Evaluate effector competence; key for benchmarking expanded cells and post-engineering function.

Marker choice should be adapted to sample type and biological question.

#### 2.2.3 Suitability of high-dimensional panels for γδ-focused questions

High-parameter panels developed for broad immunomonitoring often include γδ T cells only as a minor component and may lack γδ subset reagents or modules that are critical for γδ-centric interpretation (e.g., Vδ1/Vδ2 discrimination, NK-receptor modules, or tissue-residency markers). We therefore summarize key design considerations and highlight representative panels in terms of their strengths and limitations for γδ T cell-focused analysis (**Table 2**).

**Table 2:** Representative high-parameter panels and their suitability for γδ T cell-focused analysis.

Panel/reference (as cited)	Platform/size	γδ subset resolution	Strengths for γδ analysis	Limitations/cautions
Cossarizza et al. (35) guidelines example	Conventional FC; 12-colors	Pan-γδ + V-chain subsetting	Accessible starting point; clear gating; suitable for routine phenotyping	Limited depth for dysfunction/cytotoxicity modules
Liechti et al. (41) OMIP-058	High-parameter conventional FC 30 colors	γδ included in broader T cell set	Rich context for unconventional T cell comparisons	Lacks γδ-centric subset/functional modules
Hertoghs et al. (42) OMIP-064	High-parameter conventional FC 27 colors	γδ + MAIT/iNKT coverage	Good innate-like lymphocyte coverage	Not designed specifically for γδ biology; subset resolution depends on included reagents
Barros-Martins et al. (47) OMIP-084	Spectral FC 28 colors	Strong γδ module	Comprehensive γδ-focused phenotypic coverage incl. maturation and receptor modules	Requires spectral platform; tissue autofluorescence and unmixing require careful QC

Suitability depends on panel intent and included γδ modules.

In the next section, we focus on *ex vivo* manipulation of γδ T cells for immunotherapy. Flow-cytometric readouts can serve as practical decision points when selecting or interpreting γδ T cell expansion protocols. For example, phosphoantigen/bisphosphonate-based stimulation is expected to preferentially expand Vγ9Vδ2 cells, whereas anti-CD3-based or lectin-based activation can yield broader products that may include Vδ1 populations. Monitoring differentiation state (e.g., CD27/CCR7 with CD45RA/RO) informs expected persistence potential, while activation/proliferation and dysfunction markers help distinguish productive expansion from overstimulation. Cytotoxicity modules (granzyme B/perforin/CD107a and NK-associated receptors such as NKG2D or CD16) provide product-level readouts of effector competence that can be compared across protocols and assessed before and after genetic modification.

## 3 *Ex vivo* manipulation of γδ T cells for immunotherapy: activation, expansion, and genetic engineering

### 3.1 Immunotherapy with γδ T cells

It has been shown that γδ T cells primarily induce antitumor responses, but also protumor responses depending on the specific context. In tumor-promoting γδ T cell responses, there is usually a potentiation of IL-17 production and its secretion, which not only promotes the proliferation of tumor cells, but also their migration (56). However, generally γδ T cell infiltrates present a favorable prognostic value in cancer patients (57). γδ T cells can induce direct tumor cell killing or indirect antitumor functions by releasing specific molecules or engaging certain receptors with which they support other immune cells (e.g. NKs, B cells, αβ T cells, DCs) in antitumor activities (58). Due to their unique properties, γδ T cells have proven to be a highly promising modality for cancer immunotherapy. Early therapeutic approaches relied on antibody-based therapeutics to selectively engage γδ T cells. One example is an antibody that engages a protein on tumor cells in an agonistic fashion and thus sensitizes them to attack via γδ T cells (59) that are currently being tested in clinical trials (27). Another approach involves Bi-specific T cell Engagers (BiTEs) that have been extensively used in αβ T cells where they usually bind CD3 on T cells and tumor antigen on target cells. In γδ T cells, such constructs are designed to engage Vγ9 on γδ T cells, and tumor antigen, such as HER2, CD123 or EGFR on tumor cells. Efficacy was demonstrated in preclinical models and now these approaches are being introduced into clinical trials (6).

The success of the CAR-T cells focused on αβ T cells made it a logical step to also endow γδ T cells with an additional tumor-specificity via CAR. Such genetically engineered γδ CAR-T cells have been explored in the context of adoptive transfer (6). Early study showed feasibility of γδ CAR-T cells targeting CD19 or GD2 (14). Later, bispecific T cells expressing polyclonal repertoire of endogenous γδ T cell receptors and introduced CD19-specific CAR demonstrated decreased leukemia burden in preclinical models (60). Another study demonstrated enhanced cytotoxicity upon engineering of Vδ1 and Vδ2 with a GD-2 targeting CAR. Intriguingly, expanded Vδ2 CAR-T cells retained the ability to take up tumor antigens and cross presented the processed peptide to responder αβ T cells, which demonstrates unique features of γδ T cells, highly relevant for cancer immunotherapy (10). Vγ9Vδ2 CAR-T cells directed against MUC1-Tn antigen were expanded from peripheral blood mononuclear cells of healthy volunteers with zoledronic acid and interleukin-2 (IL-2). CAR was introduced by lentiviral delivery. The ability to lyse tumor cells in an antigen-specific manner was demonstrated on several cell lines, with similar or stronger effects than αβ CAR-T cells. However, these γδ CAR-T cells exhibited shorter persistence and reduced cytotoxic activity, which could only be improved with the addition of IL-2, limiting their translational potential (61). In addition to Vδ2, specific protocols were developed to generate Vδ1 CAR-T cells and robust tumor control was observed in preclinical studies (62–64) which was the basis for the first in human clinical trial with an anti-CD20 allogeneic Vδ1 CAR-T cells in patients with B cell malignancies (NCT04735471). The partially invariant TCR repertoire and MHC-independent antitumor activity, activation via TCR as well as NK signaling make γδ T cells an attractive candidate for allogeneic use and preparation of “off-the-shelf” cellular products for cancer treatment and show their great potential as an effector cell for cancer immunotherapy. Clear role of γδ T cells was demonstrated in animal models, where mice lacking γδ T cells became highly susceptible to multiple regimens of cutaneous carcinogenesis (65).

To prepare a suitable cell product, a well-defined knowledge of γδ T cell activation and cultivation is necessary (17, 66). Below we summarize methods of *ex vivo* activation and expansion of γδ T cells.

### 3.2 Methods of activation and expansion

**Figure 2:**
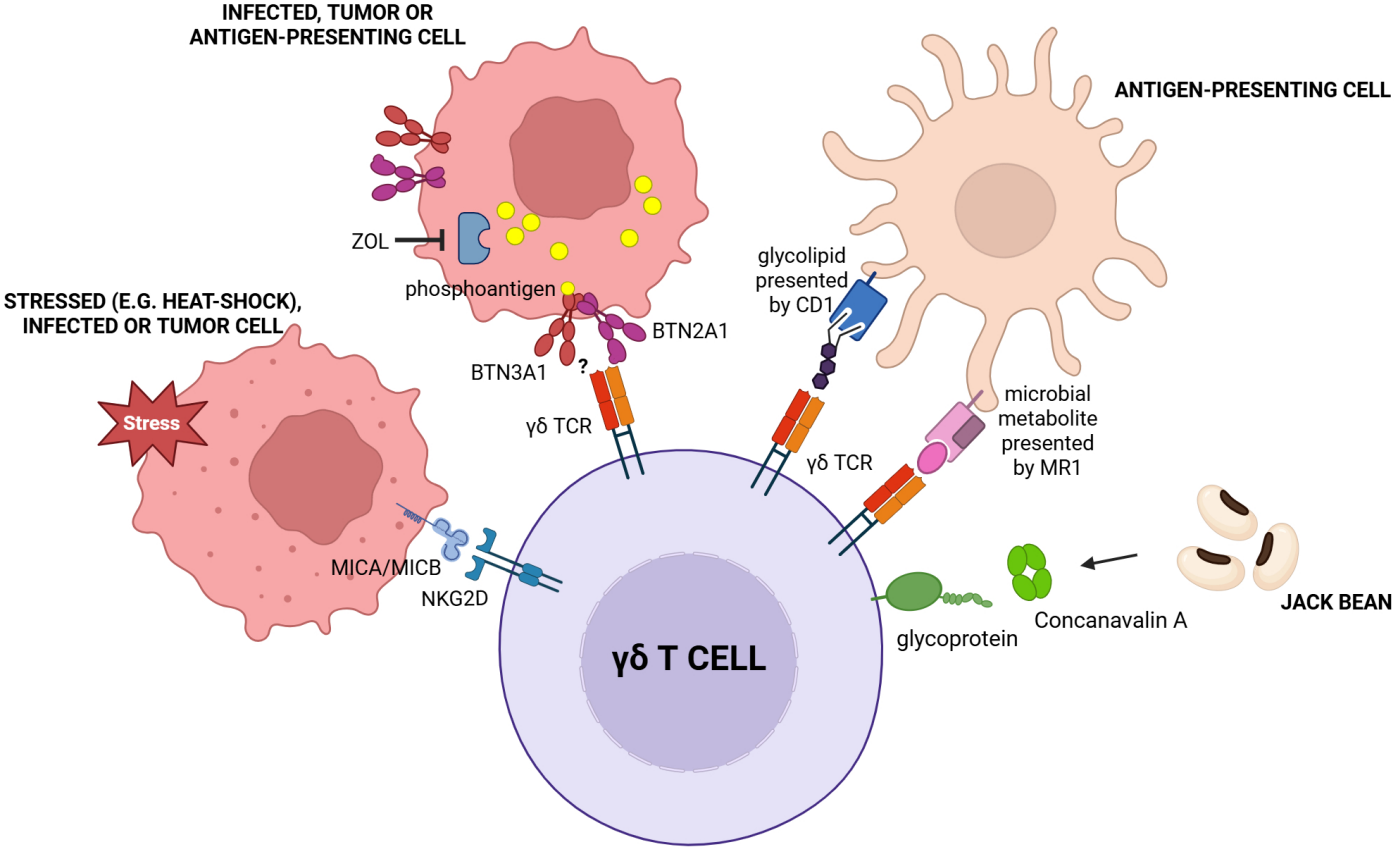
Ligands and mechanisms involved in activation of γδ T cells. Various mechanisms can activate γδ T cells, some are depicted here. Receptors NKG2D (natural killer group 2 member D) recognize MICA or MICB proteins (MHC class I chain-related protein A or B) present on cells under stress due to infection, tumor, hypoxia, heat shock etc. Certain subsets can recognize ligands presented by MHC class I-like molecules Cluster of Differentiation 1 (CD1) which present glycolipids and MHC class I-related molecule 1 (MR1) presenting microbial metabolites. Powerful mitogens such as Concanavalin A derived from Jack bean (*Canavalia ensiformis*) bind to glycoproteins on γδ T cells’ surface causing their activation and expansion. Elevated levels of phosphoantigens due to dysregulated metabolic pathway by stress or addition of zoledronate (ZOL) bind to intracellular domain of protein butyrophilin 3 A1 (BTN3A1) in tumor cell, infected cell or antigen-presenting cell. This causes conformational changes in the extracellular domain of BTN3A1 that enable association with the BTN2A1 molecules on the cell surface. It is in this state that γδ TCR can now react with BTN2A1, causing activation of γδ T cell. Created with BioRender. https://BioRender.com/1l2josj.

The complete repertoire of antigens recognized by γδ T cells and the specificity of each γδ T subset are far from being fully understood (27). Not only proteins but also other types of molecules such as lipids can be involved in recognition by γδ T cells, making their identification a challenge (7). Some of the ligands and their activation mechanisms are depicted in **Figure 2**. Some subsets of Vδ1 and Vδ3 can recognize glycolipids presented by CD1d via their TCR (67). Vγ9Vδ2 T cells recognize small phosphorylated metabolites called phosphoantigens (pAgs), albeit they do not bind directly to γδ TCRs. pAgs are generated during mevalonate pathway in mammalian cells (prototypical molecule is isopentenyl pyrophosphate; IPP) or by the non-mevalonate isoprenoid synthesis pathway in pathogens [prototypical molecule is (E)-4-hydroxy-3-methyl-but-2-enyl-pyrophosphate (HMBPP)] (6). The accumulation of HMBPP due to infection with *Mycobacterium tuberculosis* or malaria for example, triggers the same responses in γδ T cells as the accumulation of IPP (68). Intracellular pAg levels are therefore elevated under stress conditions such as infection or malignant transformation. Biphosphonates, a class of chemical compounds with two phosphonate groups, commonly used to treat osteoporosis and bone diseases, can be used to indirectly stimulate Vγ9Vδ2 γδ T cells by blocking the enzyme farnesyl pyrophosphate synthase (FPPS) in the isoprenoid biosynthesis pathway in target cells or antigen presenting cells. This leads to an accumulation of IPP and its metabolites, which react with the cytoplasmic tail of butyrophilin-3 molecules namely butyrophilin 3 A1 (BTN3A1) and induce a change in other butyrophilins detectable by the γδ TCR. BTN3A1 is essential for γδ TCR-mediated phosphoantigen recognition. The current hypothesis supported by experiments is that pAgs bind intracellularly to the B30.2 domain of BTN3A1 leading to conformational changes in the extracellular domain of BTN3A1, which are recognized by Vγ9Vδ2+ TCRs, albeit indirectly. Karunakaran et al. (69) and Rigau et al. (70) identified BTN2A1, another member of the butyrophilins, belonging to the B7 receptor family-like proteins, as the missing link. BTN2A1 therefore acts as a ligand and binds to the Vγ9-chain of γδ TCR via germline-encoded regions and without major involvement of the complementarity-determining regions (CDRs). Recapping this complex mechanism, foreign or self pAgs bind to intracellular domain of BTN3A1 in tumor cell, infected cell or antigen-presenting cell. This causes conformational changes in the extracellular domain of BTN3A1 that enable association with the BTN2A1 molecules on the cell surface of tumor, infected or antigen-presenting cell. It is in this state that γδ TCR can now react with BTN2A1, causing activation of γδ T cells (70). Zoledronate, which triggers this complex signaling, is one of the most potent bisphosphonates that has been widely used for *in vitro* and *in vivo* expansion of Vδ2 γδ T cells (68).

Since the ligands of γδ TCRs represent a broad spectrum of molecules, many of which originate from the host, and are thought to be signals of cellular stress, γδ T cells are endowed with the ability to discriminate normal homeostatic conditions from pathological ones (7). Activation of the γδ TCR must be regulated at different levels, to prevent autoimmune reactions. Co-stimulation by e.g. CD27, up- or down- regulation of ligand expression, possible conformational changes or multimerization of the ligand could all be involved in mechanisms for the discrimination of health and stress conditions via the γδ TCR (7). Mamedov et al. (71) showed that ligand abundance is not the only factor controlling the strength of γδ T cell and target cell interaction. In the case of Vγ9Vδ2 T cells they revealed that BTN2A1 and BTN3A levels at the cell surface are themselves regulated by pathways that sense the cellular metabolic state, and signal to the surveilling Vγ9Vδ2 T cells that the target cell may be transformed or stressed. The regulation of BTN3A is complex and multilayered, modulated by both conventional interferon-responsive signaling pathways as well as by metabolic stress signals (71). When cells undergo an energy crisis, AMP-activated protein kinase (AMPK) acts as a key regulator of BTN2A1 and BTN3A expression. Transformed tumor cells exhibit a combination of a hyperactive mevalonate pathway and decreased oxidative phosphorylation activity leading to cell surface upregulation of BTN2A1 and BTN3A, which, if accompanied by excess levels of pAgs, enables productive Vγ9Vδ2 TCR engagement. This mechanism allows for successful triggering of cytotoxic γδ T cells by stressed cells. Productive engagement of Vγ9Vδ2 TCRs requires both an excess level of the mevalonate pathway pAgs and sufficient expression of the BTN2A1-3A1 complex, enabled by decreased oxidative phosphorylation activity. γδ T cell surveillance allows them to detect infected or transformed cells through this combination (71).

Extensive research is being conducted to study isolation, activation, genetic engineering and expansion protocols of γδ T cells. Researchers are developing and testing different approaches for activation, including bisphosphonates that increase levels of pAgs, lectins, feeder cells or antibodies with various combinations of supporting cytokines. The protocols for successful expansion also vary in terms of possible preselection of γδ T cells or their growth in bulk with the desired cell types dominating in the end. The data is summarized in **Table 3**. Expansion fold, included in the table, is calculated by dividing final number of expanded γδ T cells (or the specific subtype) with their starting number. Current efforts are focused on finding a feasible formula, that is xeno-free and easily adaptable for an upgrade via genetic engineering such as generation of γδ CAR-T cells. Using xeno-free medium, where components are derived from the same organism without foreign species, is important for therapeutic applications, as using animal-derived components can pose additional safety risks including immune reactions towards foreign compounds. In many methods (10, 11, 34, 72–76), zoledronate, the most commonly used bisphosphonate, is utilized to increase the levels of pAgs such as IPP to activate γδ T cells. Focusing on zoledronate as an activating molecule lead to the expansion of mostly one subtype, the Vγ9Vδ2 T cells (10, 11, 34, 72–76). While zoledronate is widely used, not all donors respond equally well to zoledronate stimulation, and some do not respond at all. This has been observed in samples from healthy donors and cancer patients (72). Other methods resulted in a more heterogenous cell product. Using anti-CD3 antibodies not only Vδ2 but also Vδ1 T cells can be expanded (64, 77–81), however many of them rely on additional enrichment strategies to acquire a specific subtype Vδ1 and Vδ2 T cells can both be expanded by anti-γδ TCR antibody (82–84), plant lectins such as phytohemagglutinin (84, 85) or concanavalin A (10, 86) or proteins such as MICA (87), which are ligands of NKG2D. The latter approaches often focus on Vδ1 subtype with the help of additional enrichment strategies. Activation can also be performed with a specific anti-Vδ1 or Vδ2 antibody, resulting in a more homogenous cell product (84). Activation methods directly influence the properties of the final cell product, namely its composition. Growth factors on the other hand support the expansion process to lead to higher yield rather than to affect specific subtypes. Interleukin 2 (IL-2) is a prototypical T cell growth factor, and is therefore used in most expansion procedures (10, 11, 34, 80, 82, 83), but some reports conclude that other cytokine combinations provide better results. Wu et al. (85) achieved better results with the addition of interleukin 7 (IL-7) to a protocol containing phytohemagglutinin than with the addition of IL-2. Extensive research by Almeida et al. (77) testing 58 different activating molecules in 2488 different combinations and concentrations concluded that the highest expansion can be achieved by anti-CD3 mAb activation in the presence of IL-4 and IFN-γ, however these cells exhibited low cytotoxicity. Therefore, they developed a more complex two-step method using different cytokines without IL-2. They used IL-4, IFN-γ, IL-21, IL-1β and IL-15. Wang et al. (88) also found that IL-2 was inferior to other cytokine combinations, in their case IL-7 and IL-15. Ferry et al. (64) also obtained better results using IL-15 and anti-CD3, rather than IL-2 and anti-CD3. Cytokines and other additional growth factors present an expansion aspect worth investigating, especially when different combinations report not only higher yields but also higher cytotoxicity (77).

**Table 3:** Comparison of different methods for expansion of γδ T cells.

Source material	Starting population	Cytokines and reagents	Medium	Duration of expansion (days)	γδ T cell subtype selectivity	Expansion fold	Final purity	Final viability	Enrichment strategy	Reference
PBMC from whole blood, healthy donors	PBMC	anti-CD2, anti-CD3, IFN-γ, IL-2, IL-12	RPMI-1640 with 10% fetal bovine serum	14	Vγ9Vδ2	NR	More than 95%	More than 90%	immunomagnetic separation post expansion	Liu et al., 2005 (78)
MNC of patients with metastatic renal cell carcinoma	MNC	anti-CD3, BrHPP, IL-2	RPMI-1640 with 9% FCS	14	Vγ9Vδ2	1000	more than 70%	82%	No enrichment mentioned	Salot et al., 2007 (80)
PBMC of healthy donors and cancer patients	PBMC	IL-2, ZOL	AlyS203-gd and 10% autologous serum	14	Vγ9Vδ2	4798 ± 654 (healthy donors)	65.4% (healthy donors)	NR	No enrichment mentioned	Kondo et al., 2008 (72)
PBMC of healthy donors	Vγ9Vδ2	anti-CD3, BrHPP, feeder cells, IL-2	RPMI-1640 with 9% FCS	10	Vγ9Vδ2	~ 180 (inferred from data available)	93.8%	90.9%	MACS, pre expansion	Salot et al., 2009 (81)
PBMC of healthy donors	PBMC	IL-2, IL-18, ZOL	AlyS505N-O with 5% AB serum	14	γδ T cells	1000-5000	Over 95%	NR	MACS	Li et al., 2010 (74)
PBMC from whole blood	PBMC	IL-2, ZOL	ALyS203 or OpTmizer; autologous plasma, pooled human AB sera, or FCS (10%)	14	Vγ9	13750	93.8%	NR	No enrichment mentioned	Kondo et al., 2011 (73)
PBMC of healthy donors and acute myeloid leukemia patients	Untouched γδ T cells	IL-2, IL-15, ZOL	RPMI-1640 with 10% heat inactivated human AB serum	14	γδ T cells	Almost 1000	More than 70%	NR	Enrichment of untouched γδ T cells pre expansion	Van Acker et al., 2016 (75)
PBMC of healthy donors	PBMC	IL-2, ZOL	RPMI-1640 with 10% FBS	13-15	γδ T cells	185 (transduced); 363 (untransduced)	More than 97%	NR	αβ TCR+ cell depletion using MACS post expansion	Rozenbaum et al., 2020 (11)
PBMC of healthy donors	PBMC	IL-2, ZOL	RPMI-1640 with 10% FCS	13	Vγ9Vδ2	75	More than 70%	NR	FACS post expansion	Capsomidis et al., 2018 (10)
PBMC of healthy donors	PBMC	ConA, IL-2, IL-4	RPMI-1640 with 10% FCS	13	Vδ1 and Vδ2	50 (Vδ1); 75 (Vδ2)	Less than 10% (Vδ1); around 20% (Vδ2)	NR	FACS post expansion	Capsomidis et al., 2018 (10)
PBMC of patients with acute leukemia	PBMC	IL-2, ZOL	RPMI-1640 with 5% pooled human AB serum	10-15	Vγ9Vδ2	NR	More than 90%	NR	No enrichment mentioned	Airoldi et al., 2015 (34)
PBMC from buffy coats of healthy donors	Vδ1 and Vδ2	feeder cells, IL-2, IL-7, IL-15, PHA	IMDM with 5% heat inactivated human serum and 5% FCS	14	Vδ1 and Vδ2	380 (Vδ1); 1248 (Vδ2)	More than 99%	NR	Pre expansion MACS and FACS isolation of Vδ1 and Vδ2	Verkerk et al., 2024 (84)
PBMC from healthy donors	PBMC	IL-2, IL-15, vitC, ZOL	RPMI-1640 with 10% FBS	12-14	Vγ9Vδ2	~500 (total cell count)	More than 90%	NR	No enrichment mentioned	Xu et al., 2021 (76)
Tumour tissue from ovarian epithelial carcinoma and colonic carcinoma	Tumour tissue	IL-2, MICA	RPMI-1640 with 15% FBS	21	Vδ1	NR	62%	NR	No enrichment mentioned	Qi et al., 2003 (87)
PBMC from healthy donors or colon cancer patients	Vδ1	IL-7, PHA	RPMI-1640 with 10% FBS	14-21	Vδ1	over 100	80%	NR	Pre-expansion MACS enrichment and, FACS sorting of Vδ1	Wu et al., 2015 (85)
PBMC from whole blood or buffy coats of healthy donors or patients with chronic lymphocytic leukemia	Vδ1	anti-CD3, IL-1β, IL-4, IL-21, IL-15, IFN-γ	CTS-OpTmizer with 5% autologous plasma	21	Vδ1	up to 2,000	More than 65%	NR	MACS, pre-expansion depletion of αβ T cells, FACS sorting of Vδ1	Almeida et al., 2016 (77)
PBMC from whole blood of healthy donors	γδ T cells	ConA, feeder cells, IL-2, IL-4, PHA	AIM-V with 5% human AB serum, followed by RPMI-1640 with 10% human AB serum	21	Vδ1	More than 30	70.5 ± 4.7%	NR	Pre expansion MACS, FACS	Siegers et al., 2011 (86)
PBMC of healthy donors	PBMC	IL-7, IL-15, PTA	YM-AB	13	Vγ9Vδ2	~100 (inferred from data available)	More than 95%	NR	No enrichment mentioned	Wang et al., 2023 (88)
PBMC of lymphoma patients	PBMC	anti-γδ TCR, IL-2	RPMI-1640 with 10% FCS	14-21	Vδ1 and Vδ2	~600	~90%	NR	No enrichment mentioned	Zhou et al., 2012 (82)
PBMC from blood of lung cancer patients	PBMC	anti-γδ TCR, IL-2	RPMI-1640 with 5% pooled human AB plasma	14	γδ T cells	934	72%	NR	No enrichment mentioned	Kang et al., 2009 (83)
PBMC from healthy donor leucocyte cones	Vδ1	anti-CD3, IL-15	CTS-OpTmizer with 10% synthetic serum replacement	20	Vδ1	>1000-fold	77%	NR	MACS, pre-expansion depletion of αβ TCR- and CD56	Ferry et al., 2022 (64)

anti-CD2, anti-CD2 antibody; anti-CD3, anti-CD3 antibody; anti-γδ TCR, anti-γδ TCR antibody; BrHPP, bromohydrin pyrophosphate; ConA, concanavalin A; FACS, fluorescence-activated cell sorting; FBS, fetal bovine serum; FCS, fetal calf serum; IFN-γ, interferon γ; IL, interleukin; MACS, magnetic-activated cell sorting; MICA, MHC class I polypeptide-related sequence A; NR, not reported; PHA, phytohemagglutinin; PTA, prodrug tetrakis-pivaloyloxymethyl 2-(thiazole-2-ylamino)ethylidene-1,1-bisphosphonate; vitC, vitamin C; ZOL, zoledronate. Expansion fold is calculated by dividing final number of expanded γδ T cells (or the specific subtype) with their starting number.

High purity of the final product is key for its quality, efficacy and safety. Some report purities of 90% (34), 95% (72) or even 98% (84). For clinical use of γδ T cellular products applies the point of view, that high purity is key, whereas connection between homogeneity of composition and therapeutic efficacy is not yet clearly determined. Ferry et al. (64) debate that a Vδ1 product does not necessarily have better antitumor properties. They allow the possibility of synergistic functions of heterogeneous subtypes in the product. While heterogeneous composition could have positive effects, some researchers show potential advantages of subtype Vδ1 over Vδ2, namely longer-term preservation of functionality, greater presence in tissues and better ability to migrate into the tumor environment (10, 28, 68). Vδ2 however is known to aid in defense against malignant cells (6, 8) and is thus a logical goal of the final cell product.

Culture duration ranges between 10 (81) and 21 days (77, 82, 86, 87), with most of the protocols falling in the 14-day mark (75, 78, 80, 84, 89). Duration between 10 and 21 days leads to expected differentiation state, namely effector memory phenotype as the most common one. Articles like Zhou et al. (82) report no apparent function loss with antibody stimulated expansion taking place over three weeks, showing that even the longer span of tested duration has no negative effect on differentiation and function.

Genetically modified expanded cells maintain their functional capability. Vγ9Vδ2 subtype modified with CAR successfully recognized and lyzed tumor target cells *in vitro* (14), similar reportings being made on polyclonal γδ T cells modified with CAR (60). Comparing αβ and γδ CAR T cells shows similar cytotoxicity *in vitro*, while γδ CAR-T cells currently still fall behind *in vivo* (11). Not only Vγ9Vδ2, but also Vδ1 subtype retains functional competence after genetic modification with CAR and IL-15 construct in mouse model of human liver carcinoma, where they were able to control tumor growth (13).

Vitamin C and D’s effects on γδ T cell expansion, proliferation and cytokine production are also being investigated. Vitamin C enhances the cytokine production (notably IFN-γ), proliferative expansion and cytotoxic activity of pAg-reactive human γδ T cells (90). Xu et al. (76) found that the cells expanded with a formula including vitamin C possessed significantly improved immune effector functions, including proliferation, differentiation, and cancer cell killing, both *in vitro* and in the humanized mouse model. Vitamin D also modulates the IFN-γ secretion, proliferation and cytotoxic activity of human γδ T cells, but the reported effects are controversial (90). Several researchers have observed inhibition of proliferation, IFN-γ production and cytotoxicity (91, 92) but some studies in human cytotoxic T cells (rather than specifically in γδ T cells) report that vitamin D can reverse features of exhaustion and enhance antitumor immunity, highlighting a potentially relevant but still indirect line of evidence for γδ T cell-focused protocols (89). An interesting retrospective analysis of seasonal fluctuation of γδ T cell frequency revealed higher proportions of γδ T cells in winter compared with summer in 2625 samples of random blood donors. Investigating the correlation of taking oral vitamin D_3_ supplementation and levels of γδ T cells showed higher proportions of γδ T cells present in donors without oral vitamin D_3_ uptake, particularly in spring. However, γδ T cell frequency in blood did not directly correlate with serum levels of D_3_ metabolite (92).

## 4 Concluding remarks

γδ T cells represent a distinct and heterogeneous T cell population with subset-dependent degrees of TCR invariance and predominantly MHC-independent modes of antigen recognition and activation. They are involved in antiviral, antibacterial, antitumor, tissue-associated, and immunomodulatory responses. This diverse array of functions also suggests a role in the pathogenesis of immune-related conditions if γδ T cells are dysregulated, as they can act as either pro-inflammatory effectors or immunoregulatory cells in several systemic autoimmune diseases. Due to their distinctive properties, γδ T cells have great potential for cellular immunotherapy. Therefore, it is crucial to understand their immunobiology, phenotypic and functional characterization, and approaches for their *ex vivo* manipulation. In this review, we comprehensively summarize existing knowledge of γδ T cell immunobiology, specifying key surface markers and cytokines to evaluate their subset heterogeneity, activation states, functional and dysfunctional profiles, trafficking patterns, effector functions, and tissue-associated phenotypes. We further comprehensively integrate *ex vivo* activation, expansion, and genetic manipulation strategies, linking them to the endogenous stress-induced activation pathways that γδ T cells respond to. These biological principles underpin phosphoantigen-based stimulation, bisphosphonate-driven activation, and additional cytokine-based, antibody-based, and feeder-free methods that support the development of γδ T cell-based cellular immunotherapies. The entire repertoire of γδ T cells and their activation remains incompletely understood, and further research dedicated to advancing knowledge of their immunobiology and generating cellular products based on γδ T cells is required. This synthesis is relevant for studies of γδ T cell immunobiology and function, and for identifying phenotypes of interest in translational research aiming to inform both fundamental γδ T cell biology and the development of γδ T cell-based cellular immunotherapies.
